# Insect-Specific microRNA Involved in the Development of the Silkworm *Bombyx mori*

**DOI:** 10.1371/journal.pone.0004677

**Published:** 2009-03-05

**Authors:** Yong Zhang, Xue Zhou, Xie Ge, Jianhao Jiang, Muwang Li, Shihai Jia, Xiaonan Yang, Yunchao Kan, Xuexia Miao, Guoping Zhao, Fei Li, Yongping Huang

**Affiliations:** 1 Shanghai Institute of Plant Physiology and Ecology, Chinese Academy of Sciences, Shanghai, People's Republic of China; 2 Nanjing Agricultural University, Nanjing, Jiangsu Province, People's Republic of China; 3 Sericultural Research Institute, Chinese Academy of Agriculture Sciences, Zhengjiang, People's Republic of China; 4 Nan Yang Normal University, Nanyang, Henan Province, People's Republic of China; Columbia University, United States of America

## Abstract

MicroRNAs (miRNAs) are endogenous non-coding genes that participate in post-transcription regulation by either degrading mRNA or blocking its translation. It is considered to be very important in regulating insect development and metamorphosis. We conducted a large-scale screening for miRNA genes in the silkworm *Bombyx mori* using sequence-by-synthesis (SBS) deep sequencing of mixed RNAs from egg, larval, pupal, and adult stages. Of 2,227,930 SBS tags, 1,144,485 ranged from 17 to 25 nt, corresponding to 256,604 unique tags. Among these non-redundant tags, 95,184 were matched to the silkworm genome. We identified 3,750 miRNA candidate genes using a computational pipeline combining RNAfold and TripletSVM algorithms. We confirmed 354 miRNA genes using miRNA microarrays and then performed expression profile analysis on these miRNAs for all developmental stages. While 106 miRNAs were expressed in all stages, 248 miRNAs were egg- and pupa-specific, suggesting that insect miRNAs play a significant role in embryogenesis and metamorphosis. We selected eight miRNAs for quantitative RT-PCR analysis; six of these were consistent with our microarray results. In addition, we searched for orthologous miRNA genes in mammals, a nematode, and other insects and found that most silkworm miRNAs are conserved in insects, whereas only a small number of silkworm miRNAs has orthologs in mammals and the nematode. These results suggest that there are many miRNAs unique to insects.

## Introduction

Since miRNAs were first reported in humans, fruit flies, and nematodes, these vital participators in post-transcriptional gene regulation have received increasing attention, and many efforts have been made to discover new miRNAs in an array of organisms [1]–[6]. More than 5,000 miRNAs have been deposited in miRBase from species such as *Homo sapiens*, *Mus musculus*, *Caenorhabditis elegans*, and *Arabidopsis thaliana*, among others [7]–[8]. Recently, several miRNAs were identified in the single-celled alga *Chlamydomonas reinhardtii*, suggesting that these non-coding RNA genes have an ancient origin [9]–[10].

Because miRNAs influence the stability and translation efficiency of mRNA, they play a broad and key regulation role in many important pathways such as cellular proliferation, tumorigenesis, development, fat metabolism, behavior, embryogenesis and HIV latency [11]–[19]. In addition, abnormal expression of miRNA genes may cause human disease, dramatic phenotype changes, or death [20]. MiRNAs are also able to target several mRNA genes, and target prediction indicates that human miRNAs regulate about one-third of all mRNA genes, most of which are transcriptional and developmental factors [21].

Insects are the largest group of animals and are extremely valuable in biological and agriculture research. Insects are also important human disease vectors and agriculture pests, and efforts are necessary protect both humans and plants from disease and pest damage. Despite their importance, insects lag behind mammals, nematodes, and plants in miRNA research. At present, only 279 insect miRNAs have been identified from *Drosophila melanogaster*, *Anopheles gambiae*, *Apis mellifera*, *Bombyx mori*, and *D. pseudoobscura* in miRBase, and most of these miRNAs were computationally predicted without experimental validation. Besides *D. melanogaster* functional analysis of miRNAs has only been conducted in several insects such as *A. gambiae* and *B. mori* [22], [23].

In this study we used the mulberry silkworm, *B. mori*, which was domesticated over 5,000 years ago and is well-known for its industrial importance in sericulture. The silkworm has become a model organism for studying other lepidopteran insects that cause serious agricultural damage and is also an important model for scientific discovery in the areas of microbiology, physiology, and genetics [24]. As with all holometabolous insects, the silkworm has four distinctive developmental stages in its life cycle, including egg, larva, pupa, and adult. This makes the silkworm a good model for studying insect development and metamorphosis, which are processes that include cellular proliferation, tissue remodeling, cell migration, and programmed cell death [25]–[26]. MiRNAs play an important role in controlling the timing of post-embryonic events in other organisms. However, little is known about the functions of miRNAs in insect metamorphosis.

Here we performed a large-scale cloning of silkworm miRNAs and studied their expression profiles during development and metamorphosis. We found that most of the miRNAs were temporally expressed. Many of them were only highly expressed in the egg or pupal stages of development, suggesting that insect miRNAs play an important role in embryogenesis and larva-to-pupa metamorphosis. We also discovered that many silkworm miRNAs are conserved among insects and that only a few silkworm miRNAs have orthologs in mammals and a nematode. This suggests that there may be a set of insect-specific miRNAs.

## Results

### Sequencing-by-synthesis of silkworm small RNAs

We used sequencing-by-synthesis (SBS) technology for a large-scale sequencing of small RNAs (less than 40 nt) in the silkworm. Because miRNA is tempo-spatially expressed, we mixed the total RNAs from all developmental stages for small RNA sequencing. This procedure enabled us to detect as many small RNAs as possible with less cost. In total, we sequenced 2,227,930 tags with redundancy from the four developmental stages of the silkworm. There were 758,011 non-redundant tags ranging from 17 to 40 nt. We analyzed the size distribution of all known miRNAs and then retained all 256,604 unique SBS tags from 17 to 25 nt for further analysis. We found a total of 1,144,485 hits for the 256,604 tags, resulting in an average redundancy rate of about four. Only 95,184 tags could be matched to the silkworm genome and were used for miRNA identification.

### Computational pipeline for predicting silkworm miRNAs

**Figure 1:**
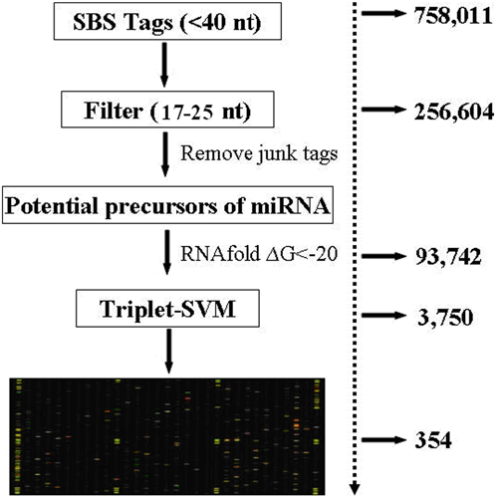
Schematic of the process from acquiring the tags through SBS to the candidate miRNAs. Total RNA from different developmental stages were pooled for SBS sequencing. Small RNAs shorter than 40 nt were excised to be sequenced. Tags with lengths ranging from 17 to 25 nt were selected for further analysis. Potential miRNA precursors were extracted from tags perfectly matched to the silkworm genomic sequence. Free energy of folding was set as ΔG<−20. Microarray assays were performed on a microfluidics chip with probes complementary to candidate miRNA sequences to confirm their existence in the silkworm.

The computational pipeline for identifying miRNA from SBS tags is shown in Figure 1. All 95,184 tags were perfectly matched to the silkworm genome, and approximately 100 nt were extracted for each match, including its flanking sequence. The secondary structure of each 100-nt fragment was predicted using RNAfold. Those fragments having a minimum free energy less than −20 were kept for evaluation using the Tri-SVM algorithm. We found 3,750 miRNA candidates that we then printed on a custom miRNA-array for validation. We identified 354 silkworm miRNAs after computational identification and microarray validation (Table S1).

### Features of silkworm miRNAs

**Figure 2:**
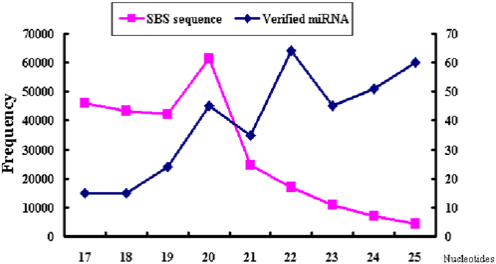
Size distribution of the 354 verified silkworm miRNAs and total SBS sequences. The sets of total SBS tags (pink) and verified miRNAs (blue) were classified for each size from 17 to 25 nt. The frequency of the SBS sequence is the left bar and the verified miRNA is the right.

**Figure 3:**
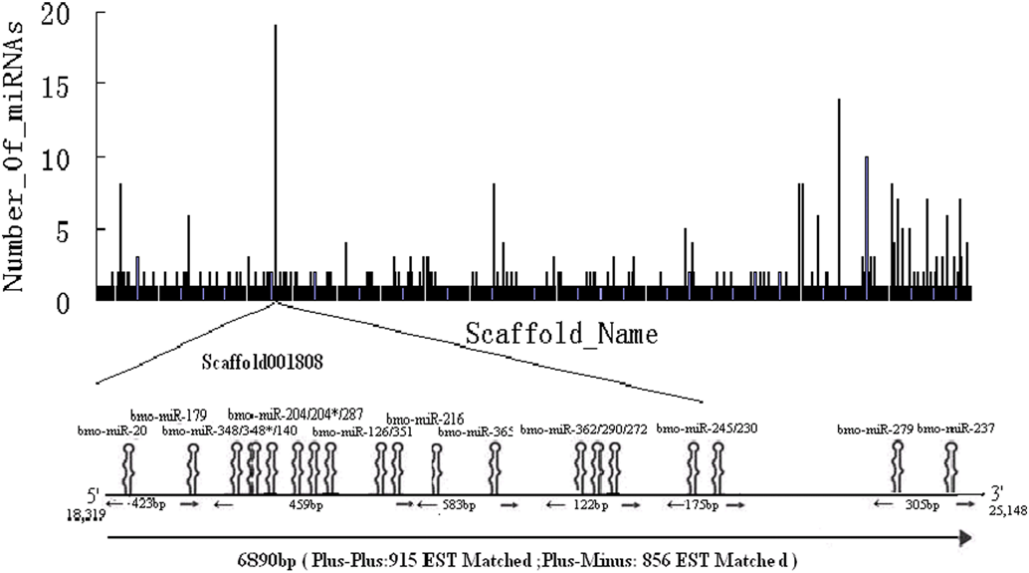
Genome-wide density analysis of the silkworm miRNAs on the scaffolds. The number of verified miRNAs was plotted on the scaffolds. A miRNA hot spot on the scaffold 001808 was shown detail both directions.

We analyzed the size distribution of the 354 identified silkworm miRNAs (Figure 2). The sequenced SBS tags were primarily 17–21 nt in length, whereas the silkworm miRNAs were mainly 22–25 nt, which is a similar length distribution of miRNAs identified in other species. This indicates that our method has no bias for abundance in raw data. All identified miRNAs were matched to scaffolds because there was no assembled silkworm genome (Figure 3). Silkworm miRNAs were not evenly distributed in the scaffolds, which is consistent with genome distribution in humans and *Drosophila*. Some scaffolds contained abundant miRNAs, such as scaffold001808. Nineteen miRNAs were located in this scaffold as a long cluster of 6,890 bp. In total, 1,771 expressed sequence tags (ESTs) matched to this region, suggesting a high expression of these miRNAs.

We classified miRNAs as a cluster if the distance between them was less than 500 bp. According to this standard, the silkworm contained 57 miRNA clusters (Table S2). Based on the annotation of protein-coding genes and miRNA positions in the scaffolds, there were 24 intronic miRNAs and nine exonic miRNAs (Table S3). The number of intronic miRNAs may have been underestimated, as many protein-coding genes are not well annotated in the silkworm.

### Insect-specific miRNAs in the silkworm

**Figure 4:**
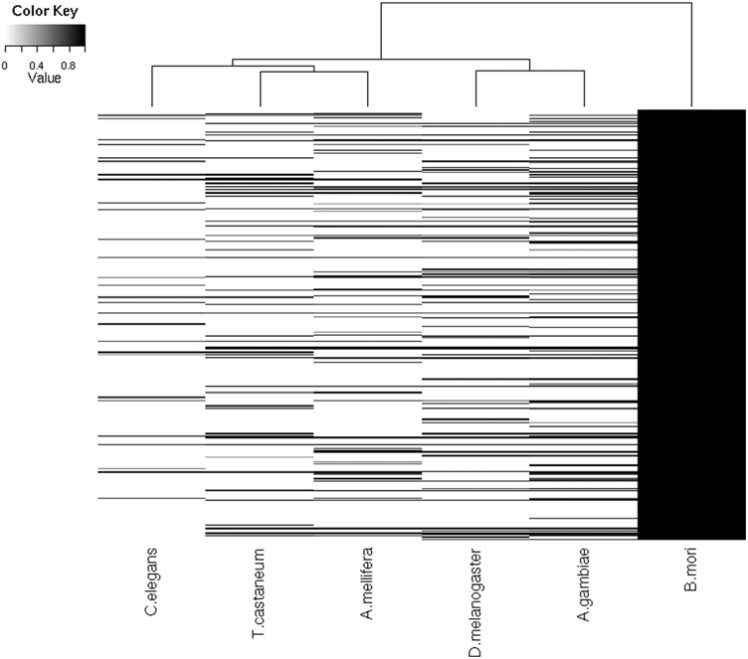
Hierarchical cluster of the homologous miRNAs. Identity of silkworm miRNAs and their homologus was used to do hierarchical cluster analysis. Different values of color key showed different sequence identity to silkworm miRNAs. The vertical direction represented the 354 silkworm miRNAs and their homologous in other organisms. The horizontal direction showed the animals we analyzed, which were *Caenorhabditis elegans*, *Tribolium ferrugineum*, *Apis mellifera*, *Drosophila melanogaster*, *Anopheles gambiae*, *Bombyx mori*.

We hypothesized that some silkworm miRNAs would be unique to insects and that if this were the case, we should find some silkworm miRNA orthologs only in insects and not in mammals nor the nematode. To test this hypothesis, we conducted a large-scale screening for orthologs in *H. sapiens*, *M. musculus*, *A. mellifera*, *A. gambiae*, *Tribolium ferrugineum*, *D. melanogaster*, and *C. elegans*. We found that only a few silkworm miRNAs were conserved among all organisms tested using miRBase. However, among the insects tested, 220 silkworm miRNAs had orthologs in *A. gambiae*, 102 in *D. melanogaster*, 114 in *A. mellifera*, and 60 in *T. ferrugineum*. Hierarchical cluster analysis shows the identity of these orthologous miRNAs and the relationship among these insects (Figure 4).

These results indicate that there are many miRNAs unique to the silkworm, but also many miRNAs specific to insects. This also explains why only a limited number of miRNAs can be identified in insects by searching for mammalian miRNA orthologs. Furthermore, these results suggest that miRNAs may be useful in phylogenetic analyses, as miRNAs follow classical phylogenetic relationships (Figure S1).

### Stage-specific silkworm miRNA expression

**Figure 5:**
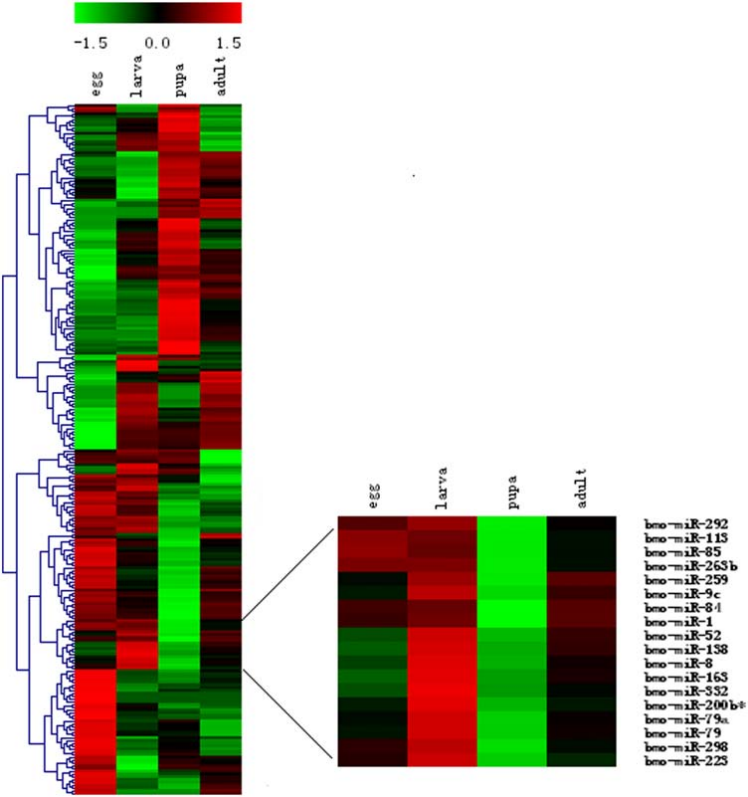
MiRNA expression profiles at different developmental stages by hierarchical clustering. Red indicates that a gene is highly expressed at the stage, whereas green indicates the opposite. Sets of miRNAs with similar patterns cluster together. Right is the enlarged image of one cluster, which express lowly in the pupa stage.

MiRNAs are important regulators in animal development. Elucidating the molecular mechanism of silkworm development is of great importance to sericulture. Thus, it is of interest to uncover the temporal expression profile of silkworm miRNAs using μParaFlo microfluidics microarrays. One-hundred and fifty silkworm miRNAs were ubiquitously expressed in all developmental stages (Figure 5). However, a majority of silkworm miRNAs were differentially expressed in the four stages (P<0.01). Hierarchical clustering of 204 stage-specific miRNAs illustrated that most miRNAs were highly expressed in either the egg or pupal stages, while only a few stage-specific miRNAs showed high expression in the larval or adult stages. This suggests that miRNAs might play an important role in embryogenesis and metamorphosis. For example, the bmo-miR-9c was normally expressed at the egg and pupae stages; however it had the highest level in larvae. This dramatic alternation of expression during these three stages signifies that bmo-miR-9c may be involved in the regulation of lava to pupae metamorphosis.

**Figure 6:**
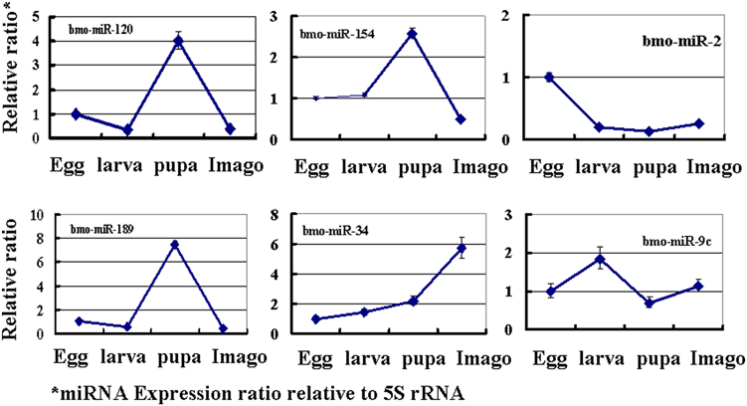
Validation of miRNAs by Quantitative real-time PCR. The transcript levels of six miRNAs at different stages were calculated relative to the amount of 5S rRNA after normalization. Each time point was replicated three times using independently collected samples. Error bar = 1 SD.

To confirm our microarray results, we performed quantitative real-time PCR analysis (Figure 6). We selected eight miRNAs that showed dramatic changes in expression level at different developmental stages. Six miRNAs showed similar expression patterns as those revealed by our microarray analysis. The expression levels of miRNAs miR-317 and miR-200b detected by qPCR were inconsistent with that of our microarray results due to unknown reasons.

## Discussion

Insect miRNAs are far less understood compared to their mammal and plant counterparts. In fact, out of the total number of miRNAs in miRBase, only 279 insect miRNAs have been reported, accounting for less than 5.5% of the total reported miRNAs. The intricacies of insect miRNAs warrant more attention not only because insects comprise the largest group of animals, but also because they have several outstanding characteristics such as high adaptability and unusual developmental processes. Insects also provide many resources to humans and also cause many medical and agricultural problems. For these reasons, identifying insect miRNAs remains an important task. Most insect miRNAs were discovered using homology searching. In this paper, we reported more than 300 silkworm miRNAs using deep sequencing. The availability of these newly reported silkworm miRNAs facilitates further functional analyses in insects.

During the preparation of this manuscript, Yu et al predicted the silkworm putative miRNAs from the genome level and cloned some miRNAs by sequencing small RNA libraries at different developmental stages [27]. We compared our miRNA dataset with theirs and found that there were 52 miRNAs in common. Furthermore, there were 272 miRNAs only found in our data (Table S5). Construction of small RNA libraries and sequencing the clones was a classic method to identify miRNAs in a specific stage. However due to the throughput of sequencing, this could only provide a partial identification of the miRNAs. The new generation of high throughput sequencing method facilitated the identification of miRNAs at the genomics level. Combined with the miRNAs array, we can validate the existence of these miRNAs and detect their expression pattern at different developmental stages. Comparison of the silkworm miRNAs in these two papers would be helpful for scientists to understand the roles of miRNAs in silkworm metamorphosis and function studies in the future.

MiRNAs are presumed to be highly conserved regulators, because they have been discovered in nearly all organisms with the exception of bacteria and fungi [28]. Our work provides a new understanding of insect miRNAs, as we have identified some insect-specific miRNAs. These miRNAs do not have orthologs in mammals and a nematode but were conserved in the insect species tested. This implies that these insect-specific miRNAs arose after the split of insects and other invertebrates. Our phylogenetic analysis demonstrated that some of these insect-specific miRNAs even appeared after the split of different insect orders, suggesting that some miRNAs have undergone dynamic evolutionary changes. miRNAs are still evolutionarily active and are undergoing a rapid “birth and death” within *Drosophila* [29], and some miRNAs even duplicate within the genome [30].

Many insect-specific proteins contribute to insect-specific phenotypes such as pheromones and metamorphosis [31]. Similarly, in this work we found insect-specific miRNAs, some of which are highly expressed in the egg or pupal developmental stages. These results imply that unique pathways of gene regulation may have evolved in insects and that these unique pathways may help us uncover the reasons why insects constitute the largest and most diverse group of animals on the planet.

MiRNAs control the timing of development in *C. elegans* and other animals [32]. In addition, miRNAs play a role in embryogenesis in mice and fruit flies [18], [33]. Moreover, miR-14 modulates the auto-regulatory loop of steroid hormone signaling via targeting on the ecdysone receptor [34]. However, the role of miRNAs in insect development and metamorphosis remains a mystery. Here, we found that many silkworm miRNAs were either egg or pupa-specific, which suggests that silkworm miRNAs function in both embryogenesis and metamorphosis. Further analysis of insect-specific miRNA expression and function would be helpful in deciphering the complex genetic network that controls insect development.

## Materials and Methods

### Silkworm

A strain of the silkworm *B. mori*, ‘p50,’ was provided by Sericultural Research Institute, Chinese Academy of Agriculture Sciences, and maintained at 25°C and a relative humidity of 70–80%. Silkworm larvae were reared at 25°C under a 12-h light/dark cycle. Five individual silkworms were sampled each day from the larval and pupal stages. Five adult moths were sampled within the first 2 days after emergence. All samples were immediately stored in liquid nitrogen.

### Cloning of silkworm miRNAs

Total RNA was isolated from silkworm eggs, larvae, pupae, and adult moths separately using Trizol (Invitrogen) according to the manufacturer's instructions. The total RNAs from different developmental stages were pooled for SBS sequencing. Cloning of the miRNAs was performed according to standard methods. About 200 µg of the total pooled RNAs were separated onto a denaturing 15% polyacrylamide gel. Small RNAs ranging from 0 to 40 nt were excised. The RNA was dephosphorylated by alkaline phosphatase (New England Biolabs) and recovered by ethanol precipitation. The small RNAs were then ligated sequentially to 5′ (5′-ACAGGUUCAGAGUUCUACAGUCCGACGAUC-3′) and 3′ (5′-UCGUAUGCCGUCUUCUGCUUG-3′) RNA adapters. The products were sequenced by Illumina Company's SBS technology [35].

### Computational pipeline for predicting miRNAs from SBS tags

Using the SBS technique, we obtained 2,227,930 tags corresponding to 758,011 unique tags from the four developmental stages of the silkworm. We selected 1,144,485 tags corresponding to 256,604 non-redundant tags, with lengths ranging from 17 to 25 nt, for further analysis. We matched these tags to the silkworm scaffolds to extract the flanking sequence as potential miRNA precursors. Only 95,184 tags could be perfectly matched to the silkworm genome sequence. We kept these tags to increase the reliability of our analysis. Two fragments of 75 bp (60+15 bp or 65+10 bp) of flanking genomic sequence around each tag were extracted, and the secondary structure and free energy was determined by using RNAfold [36]. We set the free energy of folding threshold as ΔGfolding<−20. If all four potential precursors for each tag agreed with this criterion, we chose the sequence with the lowest free energy of folding. Next, we determined the preliminary miRNA prediction using TripletSVM software [37]. Approximately 3,750 potential miRNA precursors were obtained. Microarray assays were performed on a μParaFlo microfluidics chip with each of the detection probes containing a nucleotide sequence having a coding segment complementary to a specific candidate miRNA sequence in order to confirm its existence in the silkworm. A miRNA detection signal threshold was defined as 500, which is three times the maximal background signal. We confirmed 354 candidate miRNAs, and their expression profiles were analyzed using the microarray.

### Genome location and cluster analysis

The number of small RNAs with perfect matches in either a direct or complementary strand of each scaffold was counted using the Perl program. A small RNA production hot spot in Scaffold001808 was chosen for further analysis. We determined the exact position of each miRNA within this region. We also extracted the genome sequences from Scaffold001808 according to the leftmost and rightmost matched position. We then created a local BLAST database of silkworm EST sequences. This 6,890 bp genome sequence, which contained 19 miRNA sequences, was used as a query to search local databases using the BLASTN algorithm with E-values lower than 1.0 e-100. In total, 1,771 EST sequences matched this standard and were chosen for further analysis. We statistically tested the matched EST numbers within each miRNA cluster separately that had loci with less than 500 bp.

### Searching for homologous silkworm miRNAs

The genome sequence of *D. melanogaster* was downloaded from the Berkeley Drosophila Genome Project (http://www.fruitfly.org/sequence/download.html), and the genome sequences of *A. gambiae*, *A. mellifera*, *C. elegans*, and *T. ferrugineum* were downloaded from UCSC (http://hgdownload.cse.ucsc.edu/downloads.html). Known mature human and mouse miRNA sequences were obtained from the miRBase database (http://microrna.sanger.ac.uk/cgi-bin/sequences/browse.pl). The 354 candidate silkworm miRNA seed sequences were extracted to scan the human, mouse, fruit fly, mosquito, and honeybee genomes in order to extract mature candidate 22-nt sequences using the Perl program. We then used the local program PatScan to filter these 22-nt candidate sequences allowing for one mismatch, one deletion, and one insertion with the 354 known mature miRNAs. The 22-nt homologous sequences were mapped to each species genome to extract four types of precursor sequences using the same method as above. These precursor sequences were subjected to an RNA secondary structure check using RNAfold software. If these precursors had a stable secondary structure, the sequence with lower energy was used in predictions with TripletSVM. The hierarchical cluster analysis was done by the package “gplots” of R project according to the identity of silkworm miRNAs and their homologues in other animals (http://www.r-project.org/).

### MiRNA microarray analysis

Total RNA was extracted from silkworm egg, larval, pupal, and adult samples using TRIzol reagent (Invitrogen). Five µg of total RNA from each developmental stage were size-fractionated by the mirVana kit (Ambion) and labeled with Cy3 or Cy5. Pairs of labeled samples from different stages were hybridized to dual-channel microarrays. Every stage sample was hybridized three times to another stage. Microarray assays were performed on a μParaFlo microfluidics chip with each of the detection probes containing a nucleotide sequence of coding segment complementary to a specific candidate miRNA sequence. The melting temperature of the detection probes was balanced by incorporation of a varying number of modified nucleotides with increased binding affinities. A miRNA detection signal threshold was defined as 500 after removal of the maximal signal level in the background.

### Quantitative RT-PCR expression analysis

Quantitative RT-PCR was performed using the molecular beacon technique by the Beacon Real-Time PCR Universal Reagent (Cat# GMRS-001, GenePharma, Shanghai) according to the manufacture's instructions. Primer sets for specific miRNAs, reverse transcription primer, and beacon probe are listed in Table S4. Silkworm 5s rRNA was used as a control. The total RNAs from different development days were extracted and pooled by four stages separately. For each stage, 100 ng of silkworm egg, larval, pupal, and adult total RNA were used. Quantitative RT-PCR was performed on the MX-3000P Real-Time PCR Instrument (Stratagene), and the RT-PCR conditions were 94°C for 5 min for denaturing; 50 cycles at 94°C for 15 seconds and 55°C for 30 seconds. Expression levels from each developmental stage were compared to the egg stage and statistically analyzed.

## Supporting Information

Figure S1 The phylogenetic relationships among holometabola insects. The number in the brackets shows the orthologs miRNAs found in the insect. In diptera, the two numbers are A. gambiae and D. melanogaster separately. Insect phylogeny adapted from Wheeler et al. (2001)
(1.85 MB TIF)

Table S1 Silkworm miRNA sequences.
(0.12 MB XLS)

Table S2 Position and distribution of silkworm miRNA clusters.
(0.05 MB RTF)

Table S3 Exonic and intronic miRNA.
(0.09 MB DOC)

Table S4 Primer sets used for miRNA real-time PCR.
(0.06 MB DOC)

Table S5 Comparison of silkworm miRNAs with Yu et al.
(0.14 MB XLS)
