# Supplementary figures and images for: Insect-Specific microRNA Involved in the Development of the Silkworm Bombyx mori
Source: PLoS One. 2009 Mar 5;4(3):e4677. doi: 10.1371/journal.pone.0004677 (PMC2650705; doi:10.1371/journal.pone.0004677)

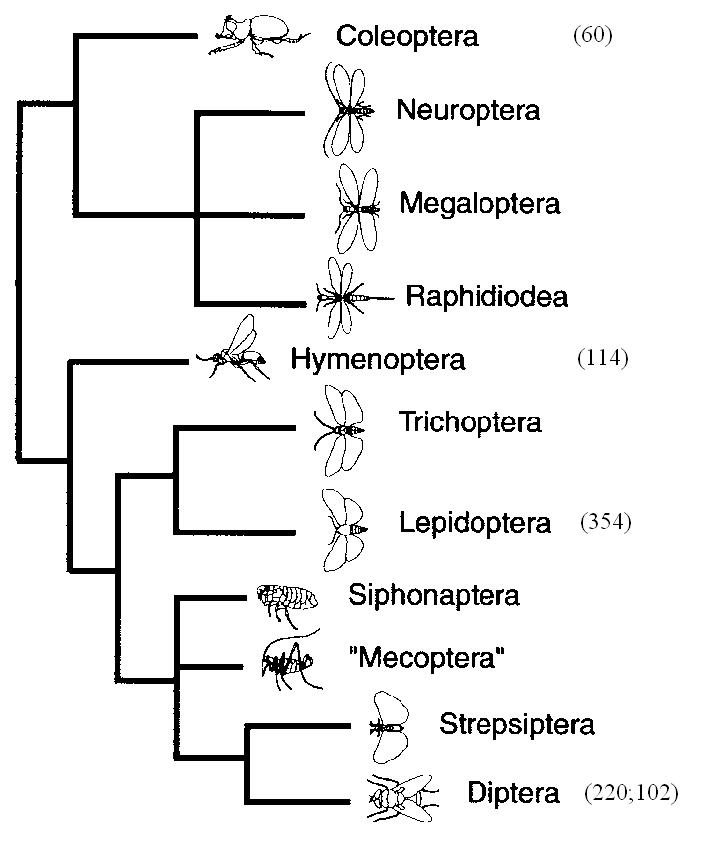

Supplement: Figure S1 — The phylogenetic relationships among holometabola insects. The number in the brackets shows the orthologs miRNAs found in the insect. In diptera, the two numbers are A. gambiae and D. melanogaster separately. Insect phylogeny adapted from Wheeler et al. (2001) (1.85 MB TIF) [file pone.0004677.s001.tif]
