# Supplementary material for: Insect-Specific microRNA Involved in the Development of the Silkworm Bombyx mori
Source: PLoS One. 2009 Mar 5;4(3):e4677. doi: 10.1371/journal.pone.0004677 (PMC2650705; doi:10.1371/journal.pone.0004677)
Supplement: Table S2 — Position and distribution of silkworm miRNA clusters. (0.05 MB RTF) [file pone.0004677.s003.rtf]

Supplementary Table S2. Position and distribution of silkworm miRNA clusters

>bmo-miR-2b|-Scaffold000226:48776-48874
GCATAGTAGCCGAGAACTCAACAAAGCTGGCTGTGATATGATCCTGAGCCCCCATGTCACAACCACCTTGTCGAGTTCTA
GCGCTACTCAACGTCGCTT
>bmo-miR-13c|-Scaffold000226:49026-49122
TATCGGTGATACGTCACACAGCCCAACTCGTCAAAAATGGCTGTGATATGAGGGTTGCTACACGACACAGCCATTTTTAC
GAGTGGGCCATGGACTG
>bmo-miR-2a|-Scaffold000226:49312-49409
TTACAGATGAAGTCGTGCTCATCAAAGCTGGCTGTGATAGTTACGTTATGACCTATGACAAACCGACTTTGATGCCACGA
CTACATTCCGCCTTGTGT
>bmo-miR-13b|-Scaffold000226:49035-49133
TACGTCACACAGCCCAACTCGTCAAAAATGGCTGTGATATGAGGGTTGCTACACGACACAGCCATTTTTACGAGTGGGCC
ATGGACTGACACGTACGAG
>bmo-miR-227|-Scaffold000226:49301-49398
CCGCGTGGTCGTTACAGATGAAGTCGTGCTCATCAAAGCTGGCTGTGATAGTTACGTTATGACCTATGACAAACCGACTT
TGATGCCACGACTACATT
>bmo-miR-2|-Scaffold000226:48896-48995
GATTCTGAAGTTCATCGCTCATCAAAGCTGGCTGTGATATGGCCAAAGTAAAAACGCATACGACAACCACTTTGTGAGCG
ACGAAACATTCGCCGCCTAG
>bmo-miR-334|-Scaffold000226:48768-48865
GGTGAAGTGCATAGTAGCCGAGAACTCAACAAAGCTGGCTGTGATATGATCCTGAGCCCCCATGTCACAACCACCTTGTC
GAGTTCTAGCGCTACTCA
>bmo-miR-111|+Scaffold001220:12698-12793
GATGAGGCAGTGAAGTTGCGTGCCGTAGGACCAGGAAGCGCGGTGAGTCGGCTGCGATGGTCCTGTTGAGGCTGCGATGC
TGGCTTGCTGTGGGAC
>bmo-miR-894a|+Scaffold001220:12877-12976
GGTTGTGCGTGTTCAAATCACGTCGGGGTCACCAGTTTGTAGGAATATACGACAAAGGGAAGATCTTCCACCGAGCTGGT
GATATTGCTCGGTAGACCGA
>bmo-miR-252a|-Scaffold001501:11476-11572
GCTGACGTCACTAAACCCTCATTGATAAGCACTTAGGCAGCAGGAGATACTACAAAACGCTCCTGCGGCACTAGTACTTA
GGAACAAGGTTTAGTTT
>bmo-miR-53|+Scaffold001789:3241-3337
TGATTCGAGCGTGAGCTTGAATGCTGCGTGCGTGTCGCCAGTGCTGCTCGAAGTTTGCAGTGAGTCGAAACTGAGCTGAA
TGCTGCGTGATTGTCGC
>bmo-miR-111|+Scaffold001789:3492-3587
GATGAGGCAGTGAAGTTGCGTGCCGTAGGACCAGGAAGCGCGGTGAGTCGGCTGCGATGGTCCTGTTGAGGCTGCGATGC
TGGCTTGCTGTGGGAC
>bmo-miR-20|+Scaffold001808:18319-18414
GGTGGGAGGATTTTGCGATGGACGCTGACGGATATCGAGTTTGCCTCAAATTTATTTTTATATCGTTGGCCTCAGATCAG
GGAGGATCACCCGCCG
>bmo-miR-126|+Scaffold001808:19230-19323
GCGTCTGTTGTCGCAGCCGTGCAGTCTCGGACTAGTGCGTGTTTGTTTGAACGTCTGCGATGATACAGTTTCGGGCACTC
GCAGGACCCGTCTT
>bmo-miR-140|+Scaffold001808:18867-18966
CTCGGGCGTACGTTTACGTGCGTTCAGATGTTGACGGAACGTTCGTTTCGTGCGTTCGTTCTCCGTCGGCACGGTACGTG
AACGTTTAACGTCCCGAGAA
>bmo-miR-179|+Scaffold001808:18649-18742
AGAGGGACGCTCCTTAGAGTCGGGTTGCTTGAGAGTGCAGCCCTAAGCGGGTGGTAAACTCCATCTAAGGCTAAATATGA
CCGCGAGACCGATA
>bmo-miR-204|+Scaffold001808:19002-19101
CGTTCGTCGTGGATTTAAGACGATCGCGCGGGAGACCGTACGAGTTTTATTCGTTTAGAACACGTTCGGACCGCGTATCG
TTCCGATCCACGCATTCGGA
>bmo-miR-216|+Scaffold001808:19402-19501
ACGAAAGTGAAGGCGAACGCTCGACGTTCGCTCAGGGAGGATGAAATTATCGAGCTACGTTCGTGATTTTCGCACTCCCG
AGGCGTCTCGTTTCCAATCA
>bmo-miR-287|+Scaffold001808:19030-19129
CGGGAGACCGTACGAGTTTTATTCGTTTAGAACACGTTCGGACCGCGTATCGTTCCGATCCACGCATTCGGACGGTATCA
TTAAACTAATCACGCACGCG
>bmo-miR-347|+Scaffold001808:19003-19103
GTTCGTCGTGGATTTAAGACGATCGCGCGGGAGACCGTACGAGTTTTATTCGTTTAGAACACGTTCGGACCGCGTATCGT
TCCGATCCACGCATTCGGACG
>bmo-miR-348|+Scaffold001808:18866-18965
TCTCGGGCGTACGTTTACGTGCGTTCAGATGTTGACGGAACGTTCGTTTCGTGCGTTCGTTCTCCGTCGGCACGGTACGT
GAACGTTTAACGTCCCGAGA
>bmo-miR-351|+Scaffold001808:19230-19325
GCGTCTGTTGTCGCAGCCGTGCAGTCTCGGACTAGTGCGTGTTTGTTTGAACGTCTGCGATGATACAGTTTCGGGCACTC
GCAGGACCCGTCTTGA
>bmo-miR-355|+Scaffold001808:18866-18965
TCTCGGGCGTACGTTTACGTGCGTTCAGATGTTGACGGAACGTTCGTTTCGTGCGTTCGTTCTCCGTCGGCACGGTACGT
GAACGTTTAACGTCCCGAGA
>bmo-miR-365|+Scaffold001808:19889-19985
GCGCTGTGGGATGAACCAAACGTAGTGTTAAGGCGCCTAAAAAACGCTCATGGGACACCATGAAAGGCGTTGGTCGCTCA
TGACAGCAGGACGGTGG
>bmo-miR-230|+Scaffold001808:21494-21593
TTGTTTCATTACTTACTCGGTTGGGCGGAAGCGGTGCGCGGTCGATAATATCGGCGGGCGCACGGTGTTTCGTTCCAAGC
GTGCAGAGTGGTGACGTGGC
>bmo-miR-245|+Scaffold001808:21418-21518
TGTAAGGAGACATGAGAGGTGTAGCATAAGTGGGAGATCGTTTCGCGCGATCGTCGCTGAAAAACCACTACTTTCATTGT
TTCATTACTTACTCGGTTGGG
>bmo-miR-272|+Scaffold001808:20959-21059
TACGTTTCGGACTGGATCCGGACCCGCGTTCTCCGGCCTTCCGCGGATCTTCCTAGCCGTAAGGTCGTGTCGGTTTCGTT
TCGTGCGCGATCGGCACGATT
>bmo-miR-290|+Scaffold001808:20957-21057
CTTACGTTTCGGACTGGATCCGGACCCGCGTTCTCCGGCCTTCCGCGGATCTTCCTAGCCGTAAGGTCGTGTCGGTTTCG
TTTCGTGCGCGATCGGCACGA
>bmo-miR-362|+Scaffold001808:20937-21035
ATGACACTCGCGAAAATCGTCTTACGTTTCGGACTGGATCCGGACCCGCGTTCTCCGGCCTTCCGCGGATCTTCCTAGCC
GTAAGGTCGTGTCGGTTTC
>bmo-miR-176|+Scaffold001980:40284-40378
GAGACATGAGGTTGAAAGTCGCACAGTCTTCGGACTGTGCTTGTTTTTGTAAGTAGATTGGACGCGCATCGCGTCTACTC
CACTCGTATTAGATA
>bmo-miR-311|+Scaffold001980:40225-40319
ATCGCGCTATGGATTTGTACGCCGGTTTATTTAAGAAAGCTGCGTAAGCATATTCGTGCGAGACATGAGGTTGAAAGTCG
CACAGTCTTCGGACT
>bmo-miR-188|+Scaffold002079:20181-20272
GGCAGCAAGGCGTTTGGCAGGCTTATACTGGAAGTCTTAATGTCGCAAACGGCAGGAACCGGCTTAGCAGGAACGAGTCA
GCAGGAACGTTG
>bmo-miR-270|+Scaffold002079:20521-20620
GTTTTTCTTTCTCACGATGTTGAAGATGGCGTTGAAGATATTTTGCAGGTTTTCTTCCTTTACGATATTGATGATGAGAG
CAGCGGCAATGAGCGCAACG
>bmo-miR-91|+Scaffold003677:22918-23016
AGCAACCCAATCTCTTGACTAACATGAGACCTACTGTGGCTGGAGCTACTAGTAGTACCATCACAATAAGTAGAAGTTAC
TTGCATTTAGTTAGGGGAG
>bmo-miR-232|+Scaffold003677:22919-23018
GCAACCCAATCTCTTGACTAACATGAGACCTACTGTGGCTGGAGCTACTAGTAGTACCATCACAATAAGTAGAAGTTACT
TGCATTTAGTTAGGGGAGGA
>bmo-miR-10a|+Scaffold004277:11119-11217
TTAGTGCCCTACATCTACCCTGTAGATCCGAATTTGTTTGAAGTGAGGCGACAAATTCGGTTCTAGAGAGGTTTGTGTGG
TGCACGCACTGCAACATTC
>bmo-miR-239|+Scaffold004277:11110-11208
GCATCATTATTAGTGCCCTACATCTACCCTGTAGATCCGAATTTGTTTGAAGTGAGGCGACAAATTCGGTTCTAGAGAGG
TTTGTGTGGTGCACGCACT
>bmo-miR-92b|+Scaffold004634:3327-3424
CGGTCGTTCACTGCGGTGGCCACGCTTAGGACGCGATTAGGTGTAAACGTTTGTGTTTCATATCAAATTGCACCAATCCC
GGCCTGCCTGTGGCCACA
>bmo-miR-92a|+Scaffold004634:3162-3259
TTTGTCCGGGAGGCCCGTTTGGCTGGGCAGTGACTGGCGCCATATTCGGTACGTGTGCGATATTGCACCAGTCCCGGCCT
ATCCGAGCGGGCCCGTCC
>bmo-miR-286|-Scaffold004926:9378-9475
AAATGGGCTTCGACGTCGCGTCTCGCGTTTTGAATGCGAAACGCCACTAGTAAAGTCACATTGCTTAGAGCCTCCCGACT
CTCGGGGCTCCACAGTAA
>bmo-miR-331|-Scaffold004926:9469-9566
ACAGTAAGCATATCTTTGCCGGATTCGGCTAGGCTGGCTTCGGCCTTAGAGGCGTTCAGGCCTAATCCCGCGGATGGTAG
CTTCGCACCACCGGCTGC
>bmo-miR-91|+Scaffold005081:12178-12276
AACAACCAAATCTCTTGACTAACATGAGACCTACTGTGGCTGGAGCTACTAGTAGTAGCATCACAATAAGTAGAAGTCAC
TTGCATTTAGTTAGGGGAG
>bmo-miR-327|+Scaffold005081:12184-12283
CAAATCTCTTGACTAACATGAGACCTACTGTGGCTGGAGCTACTAGTAGTAGCATCACAATAAGTAGAAGTCACTTGCAT
TTAGTTAGGGGAGGATGACT
>bmo-miR-33|-Scaffold006264:14897-14994
GGTGATCTCTCTATCACAATCATCATGCACCCAGTGATATAATGTTACACATATCAACTGAGAGTGTGATGACTGTATTA
GAGAGAAATAGAAGAGAT
>bmo-miR-119|-Scaffold006264:14272-14369
TAACTGTCTATTCACAATAGTTATTACATTCCCAGTGACAGAATTTGGAAACATGTCGCCTGAGGAAGTAACAACTGTTG
TTTATAGACATTGCAGAT
>bmo-miR-154|-Scaffold006264:15391-15488
GATGAATCTCTCTACCACAATCATCATACACCCAGTGATAGATTTCCACATATCAACTCAGAGTATGATGACTGCATTAG
AGAGAGAAAGACAGATAG
>bmo-miR-273|-Scaffold006264:14772-14869
GATGATCTCTCTACCACGATCATCATGCACCCAGTGATAGAATGTTACACATATCAACTGAGAGTATGATGACTGCATTA
GAGAGAGATATAGAAGAG
>bmo-miR-177|-Scaffold006264:15150-15247
TCTCTGTACCACAATCTTCATACACCCAGTGATAGAATGTCAAACATATCAACTGTGAGTATGATGACTGCATTACAGAG
AGACAGGAGAGATGTCAC
>bmo-miR-206|-Scaffold006264:11784-11880
AAAGGTCTATTCAGAATATCTATTACATTCCCAGTGACAAATCGTGAAACATGTCTCCTGGAGAAGTAATAACTGTTGTT
GAAAGACAGTGAAGACA
>bmo-miR-261|-Scaffold006264:11300-11398
TAATTTTGGAAATAGCTATTACATTCCCAGTGACAGAATGTGAATGTCTTCTGGAGAAGTAATAGCTATTGTTGAAAGAC
ATTATAGACAGTCCAGCAA
>bmo-miR-108|+Scaffold006662:18289-18385
CGACTGAACAGCGAGTGATGAGATCGGATATGAATCGTTGTGATTAATTTCCGCTCGCTCGTAGGTCACCTATCGCAGAC
TTGCCTGTCGAATATCT
>bmo-miR-136|+Scaffold006662:18272-18369
AAGGGTCTTAACTGACTCGACTGAACAGCGAGTGATGAGATCGGATATGAATCGTTGTGATTAATTTCCGCTCGCTCGTA
GGTCACCTATCGCAGACT
>bmo-miR-125|-Scaffold006662:17404-17502
GGTTAGCTAACCATTGCCTGCCTCACGCTCAGGATGCCGCGCCACGCCTCATCATCGTGGTTGTCGATCATTCACGTAGT
GTGAAGTTCTCGGTAAGAG
>bmo-miR-209|-Scaffold006662:17413-17509
ACCATTGCCTGCCTCACGCTCAGGATGCCGCGCCACGCCTCATCATCGTGGTTGTCGATCATTCACGTAGTGTGAAGTTC
TCGGTAAGAGCTTACTA
>bmo-miR-111|+Scaffold007866:9184-9279
GGCGTGATAAGGCAGTGAAGTTGCGTGCCGTAGGACCAGGAAGCGCGGTGAGTCGGCTGCGATGGTCCTGTTGAGGCTGC
GATGCTGGCTTGCTGT
>bmo-miR-212|+Scaffold007866:8997-9095
TGAGTCGAGGCTGAGCTTGAATGCTGCGTGATTGTCGCCAGTGCTGCTCGAAGTCTGCGGTGAGTCGAGACTGAGCTTGA
ATGTTGCGTGCGTGTCGTG
>bmo-miR-327|+Scaffold008625:314-413
CCAATCTCTTGACAAACATGAGACCTACTGTGGCTGGAGCTACTAGTAGTAGCATCACAATAAGTAGAAGTCACTTGCAT
TTAGTTAGGGGAGGATGACT
>bmo-miR-339|+Scaffold008625:349-448
GGAGCTACTAGTAGTAGCATCACAATAAGTAGAAGTCACTTGCATTTAGTTAGGGGAGGATGACTGCAAAATAGGTGATT
CTTGATGCAATGCTAAAAAG
>bmo-miR-328|+Scaffold009299:5358-5452
CGCACTAAGCCTGGTGTTAATATGGACCCCCCGAAGGAGTTTGGGGGGTGCAGGTTAACTAAGGAGGGGCGAACCGGGCC
AGGGGGGAAACCCAG
>bmo-miR-350|+Scaffold009299:5335-5431
GTGGTCCATGTTGACTGGACGTCCGCACTAAGCCTGGTGTTAATATGGACCCCCCGAAGGAGTTTGGGGGGTGCAGGTTA
ACTAAGGAGGGGCGAAC
>bmo-miR-281|+Scaffold009424:5722-5819
TTCCCGCCAGATATTCTGTTAATGAAGAGAGCTATCCGTCGACAGTATTGCTGATAAACACTGTCATGGAGTTGCTCTCT
TTATGAACGGATGCAGGC
>bmo-miR-281-2*|+Scaffold009424:5731-5831
GATATTCTGTTAATGAAGAGAGCTATCCGTCGACAGTATTGCTGATAAACACTGTCATGGAGTTGCTCTCTTTATGAACG
GATGCAGGCTCGACCGAAGAA
>bmo-miR-279|+Scaffold011765:1251-1349
ACTGTGGAGCCCCGAGAGTCGGGAGGCTCTAAACAATGTGACTTTACTAGTCGCGTTTCGCATTCAAAACGCGAGACGCG
ACGTCGAAGCCCATTTGGA
>bmo-miR-237|+Scaffold011765:1457-1556
CTTCCGTTCCTGGCGGGGTGTTGTGCTCGGTAGAGCAGCGTCGTGCTGCGATCTGTTGAGACTCAGCCCTGCGCCAGGTG
ATTCGTCCGAGGACGATGAA
>bmo-miR-230|+Scaffold011765:533-632
TTGTTTCATTACTTACTCGGTTGGGCGGAAGCGGTGCGCGGTCGATAATATCGGCGGGCGCACGGTGTTTCGTTCCAAGC
GTGCAGAGTGGTGACGTGGC
>bmo-miR-245|+Scaffold011765:457-557
TGTAAGGAGACATGAGAGGTGTAGCATAAGTGGGAGATCGTTTCGCGCGATCGTCGCTGAAAAACCACTACTTTCATTGT
TTCATTACTTACTCGGTTGGG
>bmo-miR-111|+Scaffold011881:1358-1453
GTCGAGATGAGACAGTGAAGTTGCGTGCCGTAGGACCAGGAAGCGCGGTGAGTCGGCTGCGATGGTCCTGTTGAGGCTGC
GATGCTGGCTTGCTGT
>bmo-miR-212|+Scaffold011881:1171-1269
TGAGTCGAGGCTGAGCTTGAATGCTGCGTGATTGTCGCCAGTGCTGCTCGAAGTCTGCGGTGAGTCGAGACTGAGCTTGA
ATGTTGCGTGCGTATCGTG
>bmo-miR-161|+Scaffold011942:5653-5745
ACCCAGCAGCCAAAAGTCCCCGCATCGGGCAGTAGTGGGATAGCGGCCGGGAGTGGGCGCTCAGTGACAGCCCGACCAAT
ATAATCAGACCCG
>bmo-miR-205|+Scaffold011942:5456-5553
ACTGGGAGTTTGGCACCTCCTGTAATCCGGCTACCCGGAGGTTGGGTACGGAGGATGGTTTGAGGTCGGGACTCACTGGC
GCTGAGTGGCCCATGTTG
>bmo-miR-76|+Scaffold012696:2750-2848
GCTGGCTTGCTGTGGGACGACTGTCGATGACTTGCTTTTAGCTGCGAGAATTGGGGAATTGTGGTAGCTGAAGTTCTTGA
TGCGCTGATGATGAAGTGT
>bmo-miR-352|+Scaffold012696:2741-2840
GGCTGCGATGCTGGCTTGCTGTGGGACGACTGTCGATGACTTGCTTTTAGCTGCGAGAATTGGGGAATTGTGGTAGCTGA
AGTTCTTGATGCGCTGATGA
>bmo-miR-101|+Scaffold014008:8772-8866
CAATTGTAGTAGTTTTCATTTCATCCGGGGATAACGAAAGCGATTGGTAAATTTGTTCAATGCGTCTTTCGTTTTTGCCT
GGATCCCAATTCTTC
>bmo-miR-180|+Scaffold014008:8800-8894
GGATAACGAAAGCGATTGGTAAATTTGTTCAATGCGTCTTTCGTTTTTGCCTGGATCCCAATTCTTCAAAAGGTTTTCGA
GGTGTAAAAGTAGAT
>bmo-miR-201|+Scaffold015648:6890-6983
CCGTGAGGGAAAGTTGAAAAGAACTTTGAAGAGAGAGTTCAAGAGTACGTGAAACCGTTCAGGGGTAAACCTGCGAAACT
CGAATGAACGAACG
>bmo-miR-297|+Scaffold015648:7138-7238
CGTTCGTCGTGGATTTAAGACGATCGCGCGGGAGACCGTACGAGTTTTATTCGTTTAGAACACGTTCGACCGCGTATCGT
TCCGATCCACGCATTCGGACG
>bmo-miR-347|+Scaffold015648:7139-7239
GTTCGTCGTGGATTTAAGACGATCGCGCGGGAGACCGTACGAGTTTTATTCGTTTAGAACACGTTCGACCGCGTATCGTT
CCGATCCACGCATTCGGACGG
>bmo-miR-162|-Scaffold015679:2433-2530
GCGTTCAGGCATAATCCCGCGGATGGTAGCTTCGCACCACCGGCCGCTCGGCCGAGTGCATGAACCAAATGTCCGAAACT
GCGGTTCCTCTCGTACTG
>bmo-miR-279|-Scaffold015679:2288-2386
TCCAAATGGGCTTCGACGTCGCGTCTCGCGTTTTGAATGCGAAACGCGACTAGTAAAGTCACATTGTTTAGAGCCTCCCG
ACTCTCGGGGCTCCACAGT
>bmo-miR-224|-Scaffold015679:2084-2183
ATCGTCCTCGGACGAATCACCTGGCGCAGGGCTGAGTCTCAACAGATCGCAGCACGACGCTGCTCTACCGAGCACAACAC
CCCGCCAGGAACGGAAGTCG
>bmo-miR-258|-Scaffold015679:2083-2183
CATCGTCCTCGGACGAATCACCTGGCGCAGGGCTGAGTCTCAACAGATCGCAGCACGACGCTGCTCTACCGAGCACAACA
CCCCGCCAGGAACGGAAGTCG
>bmo-miR-144|+Scaffold016251:4931-5030
TCTGGGCGTGAGCCCGCTTGGAGCCTCCGTCGGTGCAGATCTTGGTGGTAGTAGCAAATACTCCAGCGAGGCCCTGGAGG
ACTGACGTGGAGAAGGGTTT
>bmo-miR-166|+Scaffold016251:5484-5581
GTCGATAGAATAATGTAGGTAAGGGAAGTCGGCAAATTGGATCCGTAACTTCGGAATAAGGATTGGCTCTGAGGACCGGG
GCGTGTCGGGTTTGGACG
>bmo-miR-272|+Scaffold016251:5713-5813
TACGTTTCGGACTGGATCCGGACCCGCGTTCTCCGGCCTTCCGCGGATCTTCCTAGCCGTAAGGTCGTGTCGGTTTCGTT
TCGTGCGCGATCGGCACGATT
>bmo-miR-290|+Scaffold016251:5711-5811
CTTACGTTTCGGACTGGATCCGGACCCGCGTTCTCCGGCCTTCCGCGGATCTTCCTAGCCGTAAGGTCGTGTCGGTTTCG
TTTCGTGCGCGATCGGCACGA
>bmo-miR-230|+Scaffold016251:6248-6347
TTGTTTCATTACTTACTCGGTTGGGCGGAAGCGGTGCGCGGTCGATAATATCGGCGGGCGCACGGTGTTTCGTTCCAAGC
GTGCAGAGTGGTGACGTGGC
>bmo-miR-245|+Scaffold016251:6172-6272
TGTAAGGAGACATGAGAGGTGTAGCATAAGTGGGAGATCGTTTCGCGCGATCGTCGCTGAAAAACCACTACTTTCATTGT
TTCATTACTTACTCGGTTGGG
>bmo-miR-200b*|+Scaffold016584:1795-1894
CGGAGTAACGGTTCGCATCTTACCGGGCAGCATTAGAGTCCTGTCTATATTTTCTAATACTGTCAGGTAAAGATGTCGTC
CGCGCTCCACGTTCGTCATG
>bmo-miR-8|+Scaffold016584:1789-1887
ACACGACGGAGTAACGGTTCGCATCTTACCGGGCAGCATTAGAGTCCTGTCTATATTTTCTAATACTGTCAGGTAAAGAT
GTCGTCCGCGCTCCACGTT
>bmo-miR-291|-Scaffold016650:1507-1607
GTGACATTCACCAAGCAGAGTCATTGATGTATGCAGCTAAAAACGATGTAAAGGCGATAAATAGCCTTCACATAGCTGGG
CTGCAACTTTTTGTCTTTTTG
>bmo-miR-70|-Scaffold016650:978-1071
GTTCAATGATTTGCGTACCAGGTCATCCACTAAATCAATGATCAGGTATAGCTTTAATCATTGGATTAATCTCCACAGTT
AGAGAAGCCAGATC
>bmo-miR-77|+Scaffold017202:251-350
CGCATCGCGCGGGAGACCGTACGAGTTTTATTCGTTTAGAACACGTTCGGACCGCGTATCGTTCCGATCCACGCATTCGG
ACGGTATCATTAAACTAATC
>bmo-miR-126|+Scaffold017202:460-553
GCGTCTGTTGTCGCAGCCGTGCAGTCTCGGACTAGTGCGTGTTTGTTTGAACGTCTGCGATGATACAGTTTCGGGCACTC
GCAGGACCCGTCTT
>bmo-miR-216|+Scaffold017202:632-731
ACGAAAGTGAAGGCGAACGCTCGACGTTCGCTCAGGGAGGATGAAATTATCGAGCTACGTTCGTGATTTTCGCACTCCCG
AGGCGTCTCGTTTCCAATCA
>bmo-miR-329|+Scaffold017202:232-330
CGTCGTGGCATTTCAAGCACGCATCGCGCGGGAGACCGTACGAGTTTTATTCGTTTAGAACACGTTCGGACCGCGTATCG
TTCCGATCCACGCATTCGG
>bmo-miR-214|+Scaffold017202:218-317
ATCGCCGCACCGTTCGTCGTGGCATTTCAAGCACGCATCGCGCGGGAGACCGTACGAGTTTTATTCGTTTAGAACACGTT
CGGACCGCGTATCGTTCCGA
>bmo-miR-351|+Scaffold017202:460-555
GCGTCTGTTGTCGCAGCCGTGCAGTCTCGGACTAGTGCGTGTTTGTTTGAACGTCTGCGATGATACAGTTTCGGGCACTC
GCAGGACCCGTCTTGA
>bmo-miR-144|+Scaffold017202:1405-1504
TCTGGGCGTGAGCCCGCTTGGAGCCTCCGTCGGTGCAGATCTTGGTGGTAGTAGCAAATACTCCAGCGAGGCCCTGGAGG
ACTGACGTGGAGAAGGGTTT
>bmo-miR-365|+Scaffold017202:1119-1215
GCGCTGTGGGATGAACCAAACGTAGTGTTAAGGCGCCTAAAAAACGCTCATGGGACACCATGAAAGGCGTTGGTCGCTCA
TGACAGCAGGACGGTGG
>bmo-miR-230|+Scaffold017202:2723-2822
TTGTTTCATTACTTACTCGGTTGGGCGGAAGCGGTGCGCGGTCGATAATATCGGCGGGCGCACGGTGTTTCGTTCCAAGC
GTGCAGAGTGGTGACGTGGC
>bmo-miR-245|+Scaffold017202:2647-2747
TGTAAGGAGACATGAGAGGTGTAGCATAAGTGGGAGATCGTTTCGCGCGATCGTCGCTGAAAAACCACTACTTTCATTGT
TTCATTACTTACTCGGTTGGG
>bmo-miR-272|+Scaffold017202:2188-2288
TACGTTTCGGACTGGATCCGGACCCGCGTTCTCCGGCCTTCCGCGGATCTTCCTAGCCGTAAGGTCGTGTCGGTTTCGTT
TCGTGCGCGATCGGCACGATT
>bmo-miR-290|+Scaffold017202:2186-2286
CTTACGTTTCGGACTGGATCCGGACCCGCGTTCTCCGGCCTTCCGCGGATCTTCCTAGCCGTAAGGTCGTGTCGGTTTCG
TTTCGTGCGCGATCGGCACGA
>bmo-miR-362|+Scaffold017202:2166-2264
ATGACACTCGCGAAAATCGTCTTACGTTTCGGACTGGATCCGGACCCGCGTTCTCCGGCCTTCCGCGGATCTTCCTAGCC
GTAAGGTCGTGTCGGTTTC
>bmo-miR-279|+Scaffold018238:517-615
ACTGTGGAGCCCCGAGAGTCGGGAGGCTCTAAACAATGTGACTTTACTAGTCGCGTTTCGCATTCAAAACGCGAGACGCG
ACGTCGAAGCCCATTTGGA
>bmo-miR-237|+Scaffold018238:723-822
CTTCCGTTCCTGGCGGGGTGTTGTGCTCGGTAGAGCAGCGTCGTGCTGCGATCTGTTGAGACTCAGCCCTGCGCCAGGTG
ATTCGTCCGAGGACGATGAA
>bmo-miR-53|+Scaffold018275:3843-3939
TGAGTCGAGGTTGAGCTTGAATGCTGCGTGCGTGTCGCCAGTGCTGCTCGAAGTTTGCAGTGAGTCGAAACTGAGCTGAA
TGCTGCGTGATTGTCGC
>bmo-miR-111|+Scaffold018275:4089-4184
GGCGTGATGAGGCAGTGAAGTTGCGTGCCGTAGGACCAGGAAGCGCGGTGAGTCGGCTGCGATGGTCCTGTTGAGGCTGC
GATGCTGGCTTGATGT
>bmo-miR-126|+Scaffold018288:4384-4477
GCGTCTGTTGTCGCAGCCGTGCAGTCTCGGACTAGTGCGTGTTTGTTTGAACGTCTGCGATGATACAGTTTCGGGCACTC
GCAGGACCCGTCTT
>bmo-miR-140|+Scaffold018288:4021-4120
CTCGGGCGTACGTTTACGTGCGTTCAGATGTTGACGGAACGTTCGTTTCGTGCGTTCGTTCTCCGTCGGCACGGTACGTG
AACGTTTAACGTCCCGAGAA
>bmo-miR-204|+Scaffold018288:4156-4255
CGTTCGTCGTGGATTTAAGACGATCGCGCGGGAGACCGTACGAGTTTTATTCGTTTAGAACACGTTCGGACCGCGTATCG
TTCCGATCCACGCATTCGGA
>bmo-miR-216|+Scaffold018288:4556-4655
ACGAAAGTGAAGGCGAACGCTCGACGTTCGCTCAGGGAGGATGAAATTATCGAGCTACGTTCGTGATTTTCGCACTCCCG
AGGCGTCTCGTTTCCAATCA
>bmo-miR-287|+Scaffold018288:4184-4283
CGGGAGACCGTACGAGTTTTATTCGTTTAGAACACGTTCGGACCGCGTATCGTTCCGATCCACGCATTCGGACGGTATCA
TTAAACTAATCACGCACGCG
>bmo-miR-300|+Scaffold018288:3990-4088
GAATGAACGAACGGAGAGATCATCGTTTTCTCTCGGGCGTACGTTTACGTGCGTTCAGATGTTGACGGAACGTTCGTTTC
GTGCGTTCGTTCTCCGTCG
>bmo-miR-347|+Scaffold018288:4157-4257
GTTCGTCGTGGATTTAAGACGATCGCGCGGGAGACCGTACGAGTTTTATTCGTTTAGAACACGTTCGGACCGCGTATCGT
TCCGATCCACGCATTCGGACG
>bmo-miR-348|+Scaffold018288:4020-4119
TCTCGGGCGTACGTTTACGTGCGTTCAGATGTTGACGGAACGTTCGTTTCGTGCGTTCGTTCTCCGTCGGCACGGTACGT
GAACGTTTAACGTCCCGAGA
>bmo-miR-351|+Scaffold018288:4384-4479
GCGTCTGTTGTCGCAGCCGTGCAGTCTCGGACTAGTGCGTGTTTGTTTGAACGTCTGCGATGATACAGTTTCGGGCACTC
GCAGGACCCGTCTTGA
>bmo-miR-355|+Scaffold018288:4020-4119
TCTCGGGCGTACGTTTACGTGCGTTCAGATGTTGACGGAACGTTCGTTTCGTGCGTTCGTTCTCCGTCGGCACGGTACGT
GAACGTTTAACGTCCCGAGA
>bmo-miR-91|+Scaffold018356:499-597
AACAACCCAATCTCTTGACTAACATGAGACCTACTGTGGCTGGAGCTACTAGTAGTAGCATCACAATAAGTAGAAGTCAC
TTGCATTTAGTTAGGGGAG
>bmo-miR-327|+Scaffold018356:500-599
ACAACCCAATCTCTTGACTAACATGAGACCTACTGTGGCTGGAGCTACTAGTAGTAGCATCACAATAAGTAGAAGTCACT
TGCATTTAGTTAGGGGAGGA
>bmo-miR-339|+Scaffold018356:540-639
GGAGCTACTAGTAGTAGCATCACAATAAGTAGAAGTCACTTGCATTTAGTTAGGGGAGGATGACTGCAAAATAGGTGATT
CTTGATGCAATGCTAAAAAG
>bmo-miR-173|+Scaffold018547:2193-2286
AGATTTTGGACTCCGGGGGGAGTATGGTTGCAAAGCTGAAACTTAAAGGAATTGACGGAAGGGCACCACCAGGAGTGGAG
CCTGCGGCTTAATT
>bmo-miR-194|+Scaffold018547:2146-2242
CGCCGACGTTACTACAATGGCTCGGCGGGCAGCTTCCGGGAAACCAAAGATTTTGGACTCCGGGGGGAGTATGGTTGCAA
AGCTGAAACTTAAAGGA
>bmo-miR-237|+Scaffold019106:2185-2284
CTTCCGTTCCTGGCGGGGTGTTGTGCTCGGTAGAGCAGCGTCGTGCTGCGATCTGTTGAGACTCAGCCCTGCGCCAGGTG
ATTCGTCCGAGGACGATGAA
>bmo-miR-279|+Scaffold019106:1979-2077
ACTGTGGAGCCCCGAGAGTCGGGAGGCTCTAAACAATGTGACTTTACTAGTCGCGTTTCGCATTCAAAACGCGAGACGCG
ACGTCGAAGCCCATTTGGA
>bmo-miR-230|+Scaffold019106:1261-1360
TTGTTTCATTACTTACTCGGTTGGGCGGAAGCGGTGCGCGGTCGATAATATCGGCGGGCGCACGGTGTTTCGTTCCAAGC
GTGCAGAGTGGTGACGTGGC
>bmo-miR-245|+Scaffold019106:1185-1285
TGTAAGGAGACATGAGAGGTGTAGCATAAGTGGGAGATCGTTTCGCGCGATCGTCGCTGAAAAACCACTACTTTCATTGT
TTCATTACTTACTCGGTTGGG
>bmo-miR-272|+Scaffold019106:726-826
TACGTTTCGGACTGGATCCGGACCCGCGTTCTCCGGCCTTCCGCGGATCTTCCTAGCCGTAAGGTCGTGTCGGTTTCGTT
TCGTGCGCGATCGGCACGATT
>bmo-miR-290|+Scaffold019106:724-824
CTTACGTTTCGGACTGGATCCGGACCCGCGTTCTCCGGCCTTCCGCGGATCTTCCTAGCCGTAAGGTCGTGTCGGTTTCG
TTTCGTGCGCGATCGGCACGA
>bmo-miR-58|-Scaffold019122:2795-2894
TCGCACACATGCTAGACTCCTTGGTCCGTGTTTCAAGACGGGTCCTGCGAGTGCCCGAAACTGTATCATCGCAGACGTTC
AAACAAACACGCACTAGTCC
>bmo-miR-231|-Scaffold019122:2810-2909
ACTCCTTGGTCCGTGTTTCAAGACGGGTCCTGCGAGTGCCCGAAACTGTATCATCGCAGACGTTCAAACAAACACGCACT
AGTCCGAGACTGCACGGCTG
>bmo-miR-310|-Scaffold019122:2796-2895
CGCACACATGCTAGACTCCTTGGTCCGTGTTTCAAGACGGGTCCTGCGAGTGCCCGAAACTGTATCATCGCAGACGTTCA
AACAAACACGCACTAGTCCG
>bmo-miR-58|-Scaffold019415:3527-3626
TCGCACACATGCTAGACTCCTTGGTCCGTGTTTCAAGACGGGTCCTGCGAGTGCCCGAAACTGTATCATCGCAGACGTTC
AAACAAACACGCACTAGTCC
>bmo-miR-231|-Scaffold019415:3542-3641
ACTCCTTGGTCCGTGTTTCAAGACGGGTCCTGCGAGTGCCCGAAACTGTATCATCGCAGACGTTCAAACAAACACGCACT
AGTCCGAGACTGCACGGCTG
>bmo-miR-310|-Scaffold019415:3528-3627
CGCACACATGCTAGACTCCTTGGTCCGTGTTTCAAGACGGGTCCTGCGAGTGCCCGAAACTGTATCATCGCAGACGTTCA
AACAAACACGCACTAGTCCG
>bmo-miR-170|-Scaffold019415:443-537
CAACACCCCGCCAGGAACGGAAGTCGTCTACAGACTATTCCGAGCCCCGACATCGAACTGAGTTATATCCGGACCTTCGG
AGCCGTGATGCACGT
>bmo-miR-224|-Scaffold019415:369-468
ATCGTCCTCGGACGAATCACCTGGCGCAGGGCTGAGTCTCAACAGATCGCAGCACGACGCTGCTCTACCGAGCACAACAC
CCCGCCAGGAACGGAAGTCG
>bmo-miR-258|-Scaffold019415:368-468
CATCGTCCTCGGACGAATCACCTGGCGCAGGGCTGAGTCTCAACAGATCGCAGCACGACGCTGCTCTACCGAGCACAACA
CCCCGCCAGGAACGGAAGTCG
>bmo-miR-58|-Scaffold019727:1052-1151
TCGCACACATGCTAGACTCCTTGGTCCGTGTTTCAAGACGGGTCCTGCGAGTGCCCGAAACTGTATCATCGCAGACGTTC
AAACAAACACGCACTAGTCC
>bmo-miR-231|-Scaffold019727:1067-1166
ACTCCTTGGTCCGTGTTTCAAGACGGGTCCTGCGAGTGCCCGAAACTGTATCATCGCAGACGTTCAAACAAACACGCACT
AGTCCGAGACTGCACGGCTG
>bmo-miR-310|-Scaffold019727:1053-1152
CGCACACATGCTAGACTCCTTGGTCCGTGTTTCAAGACGGGTCCTGCGAGTGCCCGAAACTGTATCATCGCAGACGTTCA
AACAAACACGCACTAGTCCG
>bmo-miR-58|-Scaffold019937:2055-2154
TCGCACACATGCTAGACTCCTTGGTCCGTGTTTCAAGACGGGTCCTGCGAGTGCCCGAAACTGTATCATCGCAGACGTTC
AAACAAACACGCACTAGTCC
>bmo-miR-187|-Scaffold019937:2529-2626
CGTAAACGTACGCCCGAGAGAAAACGATGAATCTCTCCGTTCGTTCATTCGAGTTTCGCAGGTTTACCCCTGAACGGTTT
CACGTACTCTTGAACTCT
>bmo-miR-231|-Scaffold019937:2070-2169
ACTCCTTGGTCCGTGTTTCAAGACGGGTCCTGCGAGTGCCCGAAACTGTATCATCGCAGACGTTCAAACAAACACGCACT
AGTCCGAGACTGCACGGCTG
>bmo-miR-310|-Scaffold019937:2056-2155
CGCACACATGCTAGACTCCTTGGTCCGTGTTTCAAGACGGGTCCTGCGAGTGCCCGAAACTGTATCATCGCAGACGTTCA
AACAAACACGCACTAGTCCG
>bmo-miR-175|-Scaffold020292:231-326
TCCGGTGTCCGGGCCTGGTGAGATTTCCCGTGTTGAGTCAAATTAAGCCGCAGGCTCCACTCCTGGTGGTGCCCTTCCGT
CAATTCCTTTAAGTTT
>bmo-miR-189|-Scaffold020292:330-425
CTTTGCAACCATACTCCCCCCGGAGTCCAAAATCTTTGGTTTCCCGGAAGCTGCCCGCCGAGCCATTGTAGTAACGTCGG
CGGATCGCTAGATGAC
>bmo-miR-6|-Scaffold020623:2715-2812
CGATGCATTACTAAGAGAGAAGTGCACGTCTTCTCGGGACGTTAAACGTTCACGTACCGTGCCGACGGAGAACGAACGCA
CGAAACGAACGTTCCGTC
>bmo-miR-58|-Scaffold020623:2354-2453
TCGCACACATGCTAGACTCCTTGGTCCGTGTTTCAAGACGGGTCCTGCGAGTGCCCGAAACTGTATCATCGCAGACGTTC
AAACAAACACGCACTAGTCC
>bmo-miR-169|-Scaffold020623:2678-2772
CCCGCGCGATCGTCTTAAATCCACGACGAACGGGTCGCGATGCATTACTAAGAGAGAAGTGCACGTCTTCTCGGGACGTT
AAACGTTCACGTACC
>bmo-miR-183|-Scaffold020623:2331-2427
AAAAATTTATAAAACTCAATGACTCGCACACATGCTAGACTCCTTGGTCCGTGTTTCAAGACGGGTCCTGCGAGTGCCCG
AAACTGTATCATCGCAG
>bmo-miR-231|-Scaffold020623:2369-2468
ACTCCTTGGTCCGTGTTTCAAGACGGGTCCTGCGAGTGCCCGAAACTGTATCATCGCAGACGTTCAAACAAACACGCACT
AGTCCGAGACTGCACGGCTG
>bmo-miR-310|-Scaffold020623:2355-2454
CGCACACATGCTAGACTCCTTGGTCCGTGTTTCAAGACGGGTCCTGCGAGTGCCCGAAACTGTATCATCGCAGACGTTCA
AACAAACACGCACTAGTCCG
>bmo-miR-279|+Scaffold021254:226-324
ACTGTGGAGCCCCGAGAGTCGGGAGGCTCTAAACAATGTGACTTTACTAGTCGCGTTTCGCATTCAAAACGCGAGACGCG
ACGTCGAAGCCCATTTGGA
>bmo-miR-229|+Scaffold021254:11-110
GCGTTTAGACCGTCGTGAGACAGGTTACTTTTACCCTACTGATGGCTTGTCGTTGCGATAGTAATACTGCTCAGTACGAG
AGGAACCGCAGTTTCGGACA
>bmo-miR-237|+Scaffold021254:432-531
CTTCCGTTCCTGGCGGGGTGTTGTGCTCGGTAGAGCAGCGTCGTGCTGCGATCTGTTGAGACTCAGCCCTGCGCCAGGTG
ATTCGTCCGAGGACGATGAA
>bmo-miR-50|-Scaffold021485:849-947
TCAACGCGAGCTTATGACTCGCGCTTACTAGGAATTCCTCGTTTATGGGGGATAATTGCAAACCCCAATCCCCAGCACGA
AGGAGTTTCAGCGGGTTGC
>bmo-miR-182|-Scaffold021485:1274-1372
CCGGGCCTGGTGAGATTTCCCGTGTTGAGTCAAATTAAGCCGCAGGCTCCACTCCTGGTGGTGCCCTTCCGTCAATTCCT
TTAAGTTTCAGCTTTGCAA
>bmo-miR-190|-Scaffold021900:711-806
CAGGAACGGAAGTCGTCTACAGACTATTCCGAGCCCCGACATCGAACTGAGTTATATCCGGACCTTCGGAGCCGTGATGC
ACGTGTTAAGAAAAAA
>bmo-miR-279|-Scaffold021900:830-928
TCCAAATGGGCTTCGACGTCGCGTCTCGCGTTTTGAATGCGAAACGCGACTAGTAAAGTCACATTGTTTAGAGCCTCCCG
ACTCTCGGGGCTCCACAGT
>bmo-miR-224|-Scaffold021900:626-725
ATCGTCCTCGGACGAATCACCTGGCGCAGGGCTGAGTCTCAACAGATCGCAGCACGACGCTGCTCTACCGAGCACAACAC
CCCGCCAGGAACGGAAGTCG
>bmo-miR-258|-Scaffold021900:625-725
CATCGTCCTCGGACGAATCACCTGGCGCAGGGCTGAGTCTCAACAGATCGCAGCACGACGCTGCTCTACCGAGCACAACA
CCCCGCCAGGAACGGAAGTCG
>bmo-miR-144|+Scaffold021900:1947-2046
TCTGGGCGTGAGCCCGCTTGGAGCCTCCGTCGGTGCAGATCTTGGTGGTAGTAGCAAATACTCCAGCGAGGCCCTGGAGG
ACTGACGTGGAGAAGGGTTT
>bmo-miR-365|+Scaffold021900:1661-1757
GCGCTGTGGGATGAACCAAACGTAGTGTTAAGGCGCCTAAAAAACGCTCATGGGACACCATGAAAGGCGTTGGTCGCTCA
TGACAGCAGGACGGTGG
>bmo-miR-279|-Scaffold022053:485-583
TCCAAATGGGCTTCGACGTCGCGTCTCGCGTTTTGAATGCGAAACGCGACTAGTAAAGTCACATTGTTTAGAGCCTCCCG
ACTCTCGGGGCTCCACAGT
>bmo-miR-224|-Scaffold022053:281-380
ATCGTCCTCGGACGAATCACCTGGCGCAGGGCTGAGTCTCAACAGATCGCAGCACGACGCTGCTCTACCGAGCACAACAC
CCCGCCAGGAACGGAAGTCG
>bmo-miR-258|-Scaffold022053:280-380
CATCGTCCTCGGACGAATCACCTGGCGCAGGGCTGAGTCTCAACAGATCGCAGCACGACGCTGCTCTACCGAGCACAACA
CCCCGCCAGGAACGGAAGTCG
>bmo-miR-56|+Scaffold022264:1354-1452
GTATATGAATTTTTGTCGTCTTGGTTTCGTTATTCTGTTGTTGACGTTGTATTAATCGTTATTTAATATCGACGACGACG
ACGGTGCGAAGCGAAACGT
>bmo-miR-155|+Scaffold022264:1359-1454
TGAATTTTTGTCGTCTTGGTTTCGTTATTCTGTTGTTGACGTTGTATTAATCGTTATTTAATATCGACGACGACGACGGT
GCGAAGCGAAACGTTG
>bmo-miR-21|+Scaffold022331:396-494
TAAGGCCAGCTCAGCGAGGACAGAAACCTCGCGTGGAGCAAAAGGGCAAAAGCTGGCTTGATCCAGATGTTCAGTACGCA
TAGGGACTGCGAAAGCACG
>bmo-miR-160|+Scaffold022331:660-757
TGTCACCCATCAAAAGGGAACGTGAGCTGGGTTTAGACCGTCGTGAGACAGGTTAGTTTTACCCTACTGATGGCTTGTCG
TTGCGATAGTAATACTGC
>bmo-miR-190|+Scaffold022331:970-1065
AACGCGAGACGCGACGTCGAAGCCCATTTGGATCGCGGAGATCGATGCTATCGGTTTTTTTCTTAACACGTGCATCACGG
CTCCGAAGGTCCGGAT
>bmo-miR-279|+Scaffold022331:903-1001
ACTGTGGAGCCCCGAGAGTCGGGAGGCTCTAAACAATGTGACTTTACTAGTCGCGTTTCGCATTCAAAACGCGAGACGCG
ACGTCGAAGCCCATTTGGA
>bmo-miR-230|+Scaffold022331:187-286
TTGTTTCATTACTTACTCGGTTGGGCGGAAGCGGTGCGCGGTCGATAATATCGGCGGGCGCACGGTGTTTCGTTCCAAGC
GTGCAGAGTGGTGACGTGGC
>bmo-miR-237|+Scaffold022331:1109-1208
CTTCCGTTCCTGGCGGGGTGTTGTGCTCGGTAGAGCAGCGTCGTGCTGCGATCTGTTGAGACTCAGCCCTGCGCCAGGTG
ATTCGTCCGAGGACGATGAA
>bmo-miR-245|+Scaffold022331:111-211
TGTAAGGAGACATGAGAGGTGTAGCATAAGTGGGAGATCGTTTCGCGCGATCGTCGCTGAAAAACCACTACTTTCATTGT
TTCATTACTTACTCGGTTGGG
>bmo-miR-279|-Scaffold022399:518-616
TCCAAATGGGCTTCGACGTCGCGTCTCGCGTTTTGAATGCGAAACGCGACTAGTAAAGTCACATTGTTTAGAGCCTCCCG
ACTCTCGGGGCTCCACAGT
>bmo-miR-224|-Scaffold022399:314-413
ATCGTCCTCGGACGAATCACCTGGCGCAGGGCTGAGTCTCAACAGATCGCAGCACGACGCTGCTCTACCGAGCACAACAC
CCCGCCAGGAACGGAAGTCG
>bmo-miR-258|-Scaffold022399:313-413
CATCGTCCTCGGACGAATCACCTGGCGCAGGGCTGAGTCTCAACAGATCGCAGCACGACGCTGCTCTACCGAGCACAACA
CCCCGCCAGGAACGGAAGTCG
>bmo-miR-224|-Scaffold022700:2105-2204
ATCGTCCTCGGACGAATCACCTGGCGCAGGGCTGAGTCTCAACAGATCGCAGCACGACGCTGCTCTACCGAGCACAACAC
CCCGCCAGGAACGGAAGTCG
>bmo-miR-258|-Scaffold022700:2104-2204
CATCGTCCTCGGACGAATCACCTGGCGCAGGGCTGAGTCTCAACAGATCGCAGCACGACGCTGCTCTACCGAGCACAACA
CCCCGCCAGGAACGGAAGTCG
>bmo-miR-264|-Scaffold022700:2109-2208
TCCTCGGACGAATCACCTGGCGCAGGGCTGAGTCTCAACAGATCGCAGCACGACGCTGCTCTACCGAGCACAACACCCCG
CCAGGAACGGAAGTCGTCTA
>bmo-miR-298|-Scaffold022700:2106-2206
TCGTCCTCGGACGAATCACCTGGCGCAGGGCTGAGTCTCAACAGATCGCAGCACGACGCTGCTCTACCGAGCACAACACC
CCGCCAGGAACGGAAGTCGTC
