# Supplementary material for: Insect-Specific microRNA Involved in the Development of the Silkworm Bombyx mori
Source: PLoS One. 2009 Mar 5;4(3):e4677. doi: 10.1371/journal.pone.0004677 (PMC2650705; doi:10.1371/journal.pone.0004677)
Supplement: Table S3 — Exonic and intronic miRNA. (0.09 MB DOC) [file pone.0004677.s004.doc]

Supplementary Table S3. The list of intron or exon miRNAs

| miRNA | Sequence(5’->3’) | Length | Reads | Start | End | Strand | Scaffold | Intergenic Or Intron |
| --- | --- | --- | --- | --- | --- | --- | --- | --- |
| bmo-miR-233 | GGAGGACGUGGAGGAGGGCUC | 21 | 1 | 13933 | 13953 | + | Scaffold000853 | Exon |
| bmo-miR-238 | UUUGUUCGCCCCGGCUCGUGUCG | 23 | 4 | 25298 | 25320 | - | Scaffold000909 | Intron |
| bmo-miR-358 | CCCGAGCGGUCUGAGCAAACUG | 22 | 1 | 25334 | 25355 | - | Scaffold000909 | Exon |
| bmo-miR-59 | UGACUAGAUUUUCACUUAUCCUC | 23 | 1 | 15136 | 15158 | + | Scaffold001073 | Intron |
| bmo-miR-65 | UCGCUGUUUCGCUUCGAGUAGUUCC | 25 | 1 | 18418 | 18442 | + | Scaffold001274 | Intron |
| bmo-miR-271 | UACGACGAUGCGACAAAUAUGACA | 24 | 29 | 9928 | 9951 | - | Scaffold001530 | Intron |
| bmo-miR-323 | AAGGAAGGCAGCAGACG | 17 | 1 | 38024 | 38040 | - | Scaffold001723 | Intron |
| bmo-miR-73 | UGUUUGACAUAUCAGUAGGGACCGA | 25 | 1 | 36393 | 36417 | + | Scaffold002141 | Intron |
| bmo-miR-283 | AAAUAUCAGCUGGUAAUUCUGGG | 23 | 1 | 35358 | 35380 | + | Scaffold002164 | Intron |
| bmo-miR-7 | UGGAAGACUAGUGAUUUUGUUGUU | 24 | 5 | 21749 | 21772 | + | Scaffold003542 | Intron |
| bmo-miR-285 | CCCUGCGUGUUCUACCAAGUUA | 22 | 29 | 20821 | 20842 | + | Scaffold003542 | Intron |
| bmo-miR-366 | UCCCAUCCUCGUCGCCA | 17 | 115 | 17677 | 17693 | - | Scaffold003816 | Exon |
| bmo-miR-219 | GCAGGAUGGACCGAGAGG | 18 | 1 | 27772 | 27789 | + | Scaffold003912 | Intron |
| bmo-miR-129 | GGCGGACGCGAGCUACC | 17 | 1 | 19765 | 19781 | + | Scaffold004653 | Exon |
| bmo-miR-128 | AUGCAAUACAAGAUAAUGAGCUCC | 24 | 7 | 963 | 986 | - | Scaffold006676 | Intron |
| bmo-miR-9c | UCUUUGGUAUCCUAGCUGUAGA | 22 | 13 | 12989 | 13009 | - | Scaffold007035 | Exon |
| bmo-miR-165 | CUGGUCGAGAUCGGGGGC | 18 | 1 | 6625 | 6642 | + | Scaffold007725 | Exon |
| bmo-miR-111 | UGAAGUUGCGUGCCGUAGGAC | 21 | 1 | 9199 | 9219 | + | Scaffold007866 | Intron |
| bmo-miR-212 | UGAGUCGAGACUGAGCUUGAAUGU | 24 | 1 | 9057 | 9080 | + | Scaffold007866 | Intron |
| bmo-miR-336 | UACAACCAGACACGCGGCG | 19 | 1 | 3926 | 3944 | - | Scaffold008156 | Exon |
| bmo-miR-346 | UCAGUCUUGUCGAAUGGUGGGUGA | 24 | 1 | 10584 | 10607 | + | Scaffold008466 | Intron |
| bmo-miR-164 | ACCCUGAGCGUGAGGCUGGC | 20 | 1 | 13003 | 13022 | - | Scaffold009543 | Exon |
| bmo-miR-324 | GCUGUCGCGCGAAGGAUGAGU | 21 | 1 | 10003 | 10023 | - | Scaffold011488 | Intron |
| bmo-miR-161 | GACAGCCCGACCAAUAUA | 18 | 1 | 5718 | 5735 | + | Scaffold011942 | Intron |
| bmo-miR-205 | UGAGGUCGGGACUCACUGGCGCU | 23 | 1 | 5516 | 5538 | + | Scaffold011942 | Intron |
| bmo-miR-217 | CCUCCUGUAAUCCGGCUACCC | 21 | 1 | 5471 | 5491 | + | Scaffold011942 | Intron |
| bmo-miR-295 | GGGACUCACUGGCGCUGAGUGG | 22 | 2 | 5523 | 5544 | + | Scaffold011942 | Intron |
| bmo-miR-304 | ACCGAAAACAGCAGGACACUCG | 22 | 2 | 4809 | 4830 | - | Scaffold012974 | Exon |
| bmo-miR-291 | UGCAUACAUCAAUGACUCUGCUUGG | 25 | 1 | 1517 | 1541 | - | Scaffold016650 | Intron |
| bmo-miR-70 | UUUAGUGGAUGACCUGGUA | 19 | 1 | 993 | 1011 | - | Scaffold016650 | Intron |
| bmo-miR-65 | UCGCUGUUUCGCUUCGAGUAGUUCC | 25 | 1 | 608 | 632 | + | Scaffold020590 | Intron |
| bmo-miR-122 | AAGAGUUGGAGUGUCGUCAAA | 21 | 1 | 1178 | 1198 | + | Scaffold020814 | Intron |
| bmo-miR-269 | UUAUCUAACGUCGAACCGGUCUCC | 24 | 2 | 1122 | 1145 | + | Scaffold020814 | Intron |
