# Supplementary material for: Insect-Specific microRNA Involved in the Development of the Silkworm Bombyx mori
Source: PLoS One. 2009 Mar 5;4(3):e4677. doi: 10.1371/journal.pone.0004677 (PMC2650705; doi:10.1371/journal.pone.0004677)
Supplement: Table S4 — Primer sets used for miRNA real-time PCR. (0.06 MB DOC) [file pone.0004677.s005.doc]

Supplementary Table S4. Primer sets used for miRNA real-time PCR

| **Gene** |  | **Sequence** |
| --- | --- | --- |
| 5S rRNA | F primer | CCGGTTCTCGTCCGATCA |
|  | R primer | CGGTCACCCATCCAAGTACTG |
|  | Probe | FAM- CGAAGTCAAGCAACATCGGGCGT -DABCYL |
| **bmo-bantam** | F primer | GCTCCCTGAGACCCTAA |
|  | R primer | GTGGAGGCTGCTGAA |
|  | Beacon Probe | FAM- CGTGCCCTCACAAGTTGGCACG -DABCYL |
| **bmo-miR-1** | F primer | GGACTGGAATGTAAAGAAG |
|  | R primer | caTCaGATGCgTtgCgTa |
|  | Beacon Probe | FAM-cGCaCGcGgAGCTCCATACTCGtgCg -DABCYL |
| **bmo-miR-120** | F primer | GGACCCTGGGGTGGTG |
|  | R primer | caTCaGATGCgTtgCgTa |
|  | Beacon Probe | FAM-cGCaCGcGgGATTGCCCCACGtgCg -DABCYL |
| **bmo-miR-154** | F primer | GGACTCACTGGGTGTATG |
|  | R primer | caTCaGATGCgTtgCgTa |
|  | Beacon Probe | FAM-cGCaCGcGgCACAATCATCATCGtgCg -DABCYL |
| **bmo-miR-2** | F primer | GGACTATCACAGCCAGCTT |
|  | R primer | caTCaGATGCgTtgCgTa |
|  | Beacon Probe | FAM-cGCaCGcGgCGCTCATCAAACGtgCg -DABCYL |
| **bmo-miR-200b*** | F primer | GGACCATCTTACCGGGCA |
|  | R primer | caTCaGATGCgTtgCgTa |
|  | Beacon Probe | FAM-cGCaCGgcGACTCTAATGCTGCGtgCg -DABCYL |
| **bmo-miR-189** | F primer | GGACGATCCGCCGACGTt |
|  | R primer | caTCaGATGCgTtgCgTa |
|  | Beacon Probe | FAM-cGCaCGcggTTGTAGTAACCGtgCg -DABCYL |
| **bmo-miR-317** | F primer | GGACTGAACACAGCTGGT |
|  | R primer | caTCaGATGCgTtgCgTa |
|  | Beacon Probe | FAM-cGCaCGcGgACTGAGATACCCGtgCg -DABCYL |
| **bmo-miR-34** | F primer | GGACTGGCAGTGTGGTTA |
|  | R primer | caTCaGATGCgTtgCgTa |
|  | Beacon Probe | FAM-cGCaCCcGgACAACCAGCTAAGGtgCg -DABCYL |
| **bmo-miR-9c** | F primer | GGACTCTTTGGTATCCTAG |
|  | R primer | caTCaGATGCgTtgCgTa |
|  | Beacon Probe | FAM-cGCaCGcGgTCTACAGCTACGtgCg -DABCYL |
